# Statistical models for meal-level estimation of mass and energy intake using features derived from video observation and a chewing sensor

**DOI:** 10.1038/s41598-018-37161-x

**Published:** 2019-01-10

**Authors:** Xin Yang, Abul Doulah, Muhammad Farooq, Jason Parton, Megan A. McCrory, Janine A. Higgins, Edward Sazonov

**Affiliations:** 1 Institute of Business Analytics, University of Alabama, Tuscaloosa, AL United States; 2 Department of Electrical and Computer Engineering, University of Alabama, Tuscaloosa, AL United States; 3 Department of Health Sciences, Boston University, Boston, MA United States; 4 Department of Pediatrics, University of Colorado, Anschutz Medical Campus, Denver, CO United States

## Abstract

Accurate and objective assessment of energy intake remains an ongoing problem. We used features derived from annotated video observation and a chewing sensor to predict mass and energy intake during a meal without participant self-report. 30 participants each consumed 4 different meals in a laboratory setting and wore a chewing sensor while being videotaped. Subject-independent models were derived from bite, chew, and swallow features obtained from either video observation or information extracted from the chewing sensor. With multiple regression analysis, a forward selection procedure was used to choose the best model. The best estimates of meal mass and energy intake had (mean ± standard deviation) absolute percentage errors of 25.2% ± 18.9% and 30.1% ± 33.8%, respectively, and mean ± standard deviation estimation errors of −17.7 ± 226.9 g and −6.1 ± 273.8 kcal using features derived from both video observations and sensor data. Both video annotation and sensor-derived features may be utilized to objectively quantify energy intake.

## Introduction

Accurate and objective assessment of dietary intake in free-living individuals is crucial in nutrition and health studies. Traditional methods of dietary assessment estimate include self-report methods such as food frequency questionnaires, 24-hour dietary recalls and weighed or estimated food intake records^1^. However, self-report based methods are susceptible to errors due to incorrect estimation of portions consumed, incorrect identification of food consumed^1–3^, either forgetting or intentionally not reporting some foods that were consumed, and, in the case of weighed or estimated records, interference with subjects’ normal eating behaviors^4,5^. Some studies have combined self-report with other technologies, such as audio reports^6^, photographic food records^7^, personal digital assistants^8^, and smart cards^9^, to improve the accuracy of dietary assessment. However, these methods still rely on self-report and therefore may also result in inaccurate estimation of food intake^8,10^.

In the past decade, several automatic food intake monitoring devices have been developed to address the shortcomings associated with self-report methods of assessing of dietary intake^11^. Some of the automated approaches for food intake monitoring include the use of acoustic sensors^12^, piezoelectric sensors, impedance measurements^13^, motion sensors^14^, cameras^15^ and hand gesture sensors^16^. These sensors enable monitoring of different aspects of eating such as bites, chews, swallows, and hand-to-mouth gestures. Swallowing and chewing patterns are dependent on the rheological properties and textures of the foods being consumed^17^. Therefore, features derived from sensor-monitored swallowing and chewing patterns can provide useful information about the food properties. In addition, some of the sensor-based approaches have been used for estimation of mass and energy intake. For example, an acoustic-based sensor system which monitored chewing sounds and the features derived from them were used to predict bite weight for three types of foods (potato chips, lettuce, and apple) using linear regression models with prediction errors from 19.4% to 31%^18^. In^19^, a hand-gesture sensor was employed to estimate bite count, and energy intake was estimated through linear regression models and participant’s self-estimation. Mean estimation errors were −257.4 ± 790.2 kcal for participant self-estimates and 71.2 ± 562.1 kcal for the hand-gesture sensor. In^20^, the authors utilized features derived from audio sensors (zero-crossing rate, energy, spectral flux, etc.), motion sensors (zero crossing rates, temporal shape features, etc.) and food type to estimate the mass of food intake. The mean absolute percentage error was reduced from 127.3% (mean weight of food intake) to 35.4% (features included audio features, motion features, annotation features and food type). In our previous work, counts of chews and swallows were used to estimate the mass and energy content of individual food tems^21^ with the mean absolute percentage error of 32.2% ± 24.8%.

This study relies on video observation and annotation as an accepted gold standard for feeding studies^18,22,23^. Although video annotation based method is not realistic when applied to practical situations and large-scale analyses, many studies used it as a reference method^13,21,24–27^. The number bites and chews during food intake can be counted by observing jaw movement. The number of swallows can be counted by observation of the laryngopharynx during pharyngeal stage of swallowing. Our previous research showed higher than 0.95–0.98 of intra-class correlation coefficients between raters for detection and counting of bites, chews and swallows using video annotation^28^.

Sensors can provide valuable information about chewing rate and strength which is not available from video observations^18,22,23^. Features extracted from sensor signals can also provide information about the individual’s eating habits e.g. variations in chewing rate can be used to determine whether the individual is a linear or decelerated eater^29^. Similarly, swallow-related features such as swallow duration and swallowing rate can provide information about liquid and solid food swallowing^30^. Further, sensor-based features can provide information about the duration of eating during a meal^31^.

Models of mass and energy intake reported in the literature fall either under individually-calibrated or group-calibrated types. Individually-calibrated models are models built based on each participant separately. For example, in^21^, the coefficients of models to estimate the mass intake were estimated specifically for each individual. Group models are not built individually for each participant, but for all participants as a group. Examples of group models have been reported previously^19,20,32,33^. The advantage of group models is that they can be applied easily to new participants whereas individual models require building models for each new participant, which costs both time and money.

Most of the existing work on food recognition, and mass and energy intake estimation have been analyzed at the individual food item level (i.e. estimation for each food item in the meal)^21,32,33^. However, in real life, many foods are consumed simultaneously by mixing portions of several food items, therefore estimation of individual food items is less meaningful. Therefore, estimation of mass and energy intake at a meal level has more practical applications than estimation at food item level. However, very little work has been done to estimate mass intake or energy intake at the meal level.

Our long-term goal is to use sensor-based methods for objective assessment of energy intake and ingestive behavior. This study attempts to identify features of bites, chews and swallows that are most correlated to mass and energy intake and thus should be monitored by wearable sensors. The information obtained in this study is important to understand the performance of sensor based-methods and if features of the ingestion process are related to the mass and energy intake. The analysis was performed in the context of group-calibrated models of mass and energy intake for a whole meal with mixed food consumption.

## Methods

### Study population and design

The study was performed at Clarkson University. Thirty participants, 15 males and 15 females, were recruited. Participants had a mean age of 29 years (SD = 12, range = 19–58) with a mean body mass index (BMI) of 27.9 kg/m^2^ (SD = 5.5, range: 20.5–41.7). Individuals were excluded if they had difficulties of chewing and/or swallowing or had a disease associated with chewing or swallowing problems such as temporomandibular joint (TMJ) disease and dysphagia. The Institutional Review Board at Clarkson University approved the protocol, and all subjects read and signed informed consent form before participating. Data from two participants were excluded from analyses due to equipment failure.

Participants were randomly assigned to consume either breakfast, lunch, or dinner, with about one third of participants assigned to each. Participants were asked to undergo testing over four visits to the laboratory^34^. Each participant made two different meal selections from a wide variety of foods available at one of the cafeterias at the Clarkson University; one of the meals was consumed over each of three visits in order to measure intra-subject variability, and the other was consumed at the remaining visit. The order in which the meals were consumed was randomized. Overall, participants selected 110 distinct food items (both solid and liquids)^25^. The most frequently served foods included cookies, banana, apple, sandwich, salad, pasta, pizza, etc.^21^. Examples of typical foods provided are shown in Fig. S1. The food items were distributed from cafeteria and provided by Aramark Corporation, which is one of the largest food service providers in US. The ingredients of all food provided were known and available in the Aramark’s food composition database. Meals were consumed in the laboratory so that consumption could be videotaped^34^ and annotated. During the experiment, foods were kept on a scale, except for the times the participants took the food off the scale for consumption. Research staff kept a record of mass of intake for each bite by recording the food weight before and after each bite, by a commercially available scale with 1 g precision. The energy content of the consumed foods was established by a nutritionist who entered the food type and weight data into the nutritional analysis software, Nutrient Data System for Research (NDS-R, University of Minnesota, Minneapolis, MN) as described in^21^. While eating the meal, participants wore a piezoelectric strain sensor (LDT0–028K, Measurement Specialties Inc. VA, USA), which was attached below the outer ear and captured jaw movements during food intake^24,25^. The video was manually annotated by experienced research assistants (different individuals from the assistants who conducted experiments) by marking each bite, chewing bout, and swallow associated with food consumption using custom-designed software^21,34^. The use of video annotation as the gold standard for studies of eating behavior demonstrated repeatability and reproducibility of the results^13,21,25–27,35^. The research assistants also recorded information about the food physical properties (i.e. liquid or solid). All methods were performed in accordance with the relevant guidelines and regulations.

### Features extracted from video observation and sensor signal

Several features, such as numbers and time stamps of bites, chews and swallows as well as mass intake of each bite, were available directly from the video annotations (Fig. S2). Two dependent features (mass and energy intake) and fifty-seven independent features (predictors) were derived from features produced from video annotation and sensor signal. Among the independent features, fifteen features were derived from video annotation for chews, six features were derived from video annotation for bites, nine features were derived from video annotation for swallows, four features were derived from video annotation for pauses between ingestion bouts, and twenty-three features were derived from sensor signals. An illustration of the microstructure of eating including bites, chewing sequences and swallows is shown in supplemental Fig. S2.

### Dependent features

Total mass of intake for beverages and solid foods were recorded separately. Mass of intake was directly measured using a weighed food record which is the gold standard methodology. The mass of each individual food consumed was summed to calculate the total mass intake of the meal, including both beverages and solid foods. Energy intake of the meal was estimated by calculating the product of mass and energy density for the summed mass of each food. The energy density of each food item was extracted from the nutritional analysis by NDS-R^21^.

### Independent features derived from video observations

#### Bite features

Bite features derived from video observations included total bite counts per meal (*total_bite*), bite rates, and instantaneous bite frequency (*IBF*). Two different bite rate features were calculated: total bite counts divided by eating time (*avg_biteRate_Teating*) and total bite counts divided by meal time (*avg_biteRate_Tmeal*). For IBF features, we first calculated the durations between successive bite events within a meal. Then IBF was calculated by the reciprocal of each duration. Then the mean, SD, and variance of IBFs were obtained for each meal (*avg_IBF*, *sd_IBF*, and *var_IBF*).

#### Chewing features

The meal-level features included the number of chewing sequences (*chews_seq*), the total chew count in a meal (*total_chews*) and three meal-level chewing rates that were calculated as the total chew number within a meal divided by chewing duration, eating duration and meal duration respectively (*chewRate_Tchewing*, *avg_chewRate_Teating*, *avg_chewRate_Tmeal*). The features derived from individual chewing sequences included mean, SD, and variance for the following variables: number of chew counts per chewing sequence (*avg_chews_perSeq*, *sd_chews_perSeq*, *var_chews_perSeq*); duration of each chewing sequence (*avg_chews_du_perSeq*, *sd_chews_du_perSeq* and *var_chews_du_perSeq*); chewing rate for each chewing sequence (*avg_chewRate_perSeq*, *sd_chewRate_perSeq*, *var_chewRate_perSeq*).

#### Swallowing features

The meal-level swallowing features included the total swallow count per meal (*total_swallow*) and two swallowing rates that were defined as the total swallow count divided by eating duration and meal duration, respectively (*avg_swlRate_Teating* and *avg_swlRate_Tmeal*). The average, standard deviation and variance of instantaneous swallowing frequency (ISF) within a meal (*avg_ISF*, *sd_ISF*, *var_ISF*) and swallow count between successive bites (*avg_swl_bite*, *sd_swl_bite*, *var_swl_bite*) were also calculated.

#### Pause features

Pause duration within a meal was defined as the time between the consecutive ingestion events (Supplemental Fig. S2). Total pause duration (*total_pause_du*) was calculated by summing all pause durations within a meal. The average, standard deviation and variance of pause durations were also obtained (*avg_pause_du*, *sd_pause_du* and *var_pause_du*).

#### Sensor features

The Information about signal strength and chewing frequency was obtained from chewing sensor data. These features were derived from each chewing bout (based on the video annotations) of the sensor signal. Total and average values of features were computed over the meal level. These sensor features included the number of mean crossings, duration between mean crossings, entropy, waveform length, mean amplitude, periods from autocorrelation function, power of the signal and maximum frequency present in the signal spectrum. A detailed description of these features is given in supplemental Table S1.

### Statistical analysis

Using multiple linear regression analysis, two sets of models were developed, one for mass intake per meal and another for energy intake per meal derived from the weighed records as the dependent variables. Based on the type of features used in the models, five models were built: full model, bite model, chew model, swallow model and sensor model. All available features were included in the “full model”, while bite, chew, swallow, and sensor models were built by using bite, chew, swallow, and sensor features. For each model, we first used the forward selection procedure to select the most important features from the initial set of fifty-seven features using mean absolute percentage error as the criteria. The selected independent features (from one to six features per model) were then used to build multiple linear regression models with their intercepts set to 0 to predict mass intake and energy intake with a leave-one-subject-out cross-validation^36^. In this cross-validation procedure, data from the meals consumed by each participant were used as validation data whereas data from the remaining meals of 27 participants were used as the training data. The reporting errors for mass and energy were computed as follows:

$${\rm{absolute}}\,{\rm{percentage}}\,{{\rm{error}}}_{ij}=|\frac{Estimated\,valu{e}_{ij}-Actual\,valu{e}_{ij}}{Actual\,valu{e}_{ij}}\times 100 \% |,$$

where *i* is *i*th participant and *j* is *j*th meal of the *i*th participant, estimated values is the value (mass or energy intake) computed by the model, and actual value is the value (mass or energy intake) obtained from weighed food records.

The mean and SD of absolute percentage errors for each model were calculated. We also calculated the bias, limit of agreements and their corresponding 95% confidence intervals for Bland-Altman plots^37^. Bias (also called mean estimation error) was defined as mean difference between estimated mass or energy intake from models and actual mass or energy intake. Limit of agreement (LOA) were defined as bias ±1.96 SD and confidence interval for LOA was calculated as $LOA\,\pm \,{t}_{n-1}\sqrt{\frac{3S{D}^{2}}{n}}$. The 95% confidence interval for bias was calculated as $Bias\,\pm \,{t}_{n-1}\sqrt{\frac{S{D}^{2}}{n}}$^38^. Statistical analysis was conducted using SAS 9.4 (SAS institute, Cary, NC). A p-value of 0.05 was accepted as statistically significant.

## Results

### Models performance for estimating mass intake

**Figure 1:**
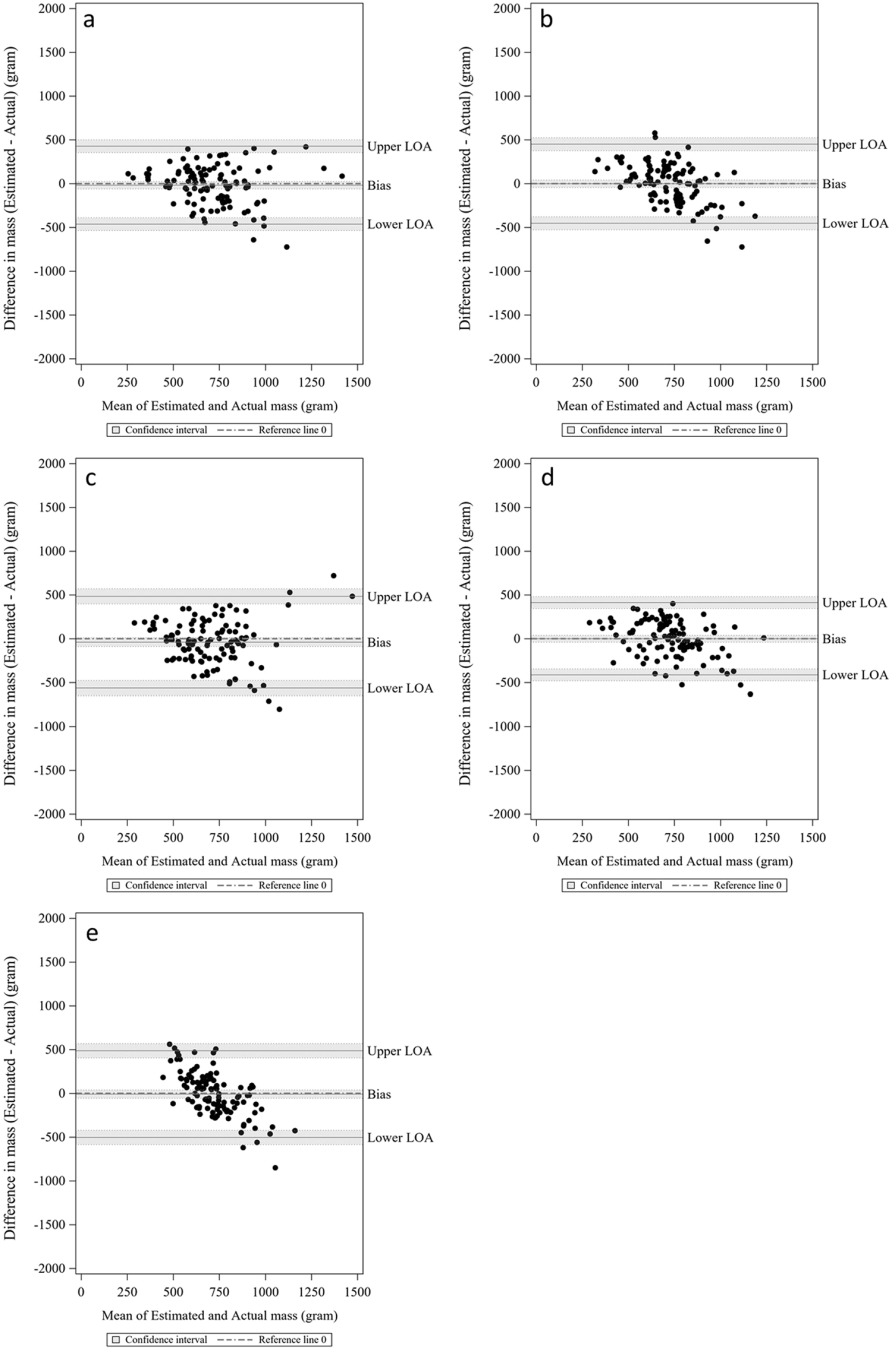
Bland-Altman plot of estimated and actual mass intake based on models. (**a**) full model; (**b**) chew model; (**c**) bite model; (**d**) swallow model; (**e**) sensor model.

Table 1 shows the mean and standard deviation of absolute percentage errors and biases for the full, chew, bite, swallow, and sensor models estimating mass intake of a meal. The full model had the lowest mean absolute percentage error (mean = 0.252, SD = 0.189). The mean and standard deviation of bias were −17.67 and 226.9 g. The Bland-Altman plots showed a LOA of −462.1 g and 427.1 g (Table 1), and the bias was not significant since the confidence interval covered 0 (Fig. 1a). Five independent features were selected to minimize mean absolute percentage error: total bite count per meal, average pause duration, variance of chew duration per chewing sequence, variance chew number per chewing sequence, and average total entropy. The total number of bites per meal has the most statistically significant coefficient (*t* value = 10.73, p < 0.0001) with about 11 g of mass intake increase for one extra bite (Table 2).

**Table 1:** Mean absolute percentage errors and mean estimation errors for mass intake models.

	Relative reporting error		Mean estimation error		Lower LOA	Upper LOA	Predictors
	Mean	SD	Mean	SD			
Full	0.252	0.189	−17.665	226.901	−462.391	427.061	total_bite avg_pause_du avg_Total_Entropy VAR_chews_du_perSeq VAR_chews_perSeq
Chew	0.291	0.282	−1.186	230.42	−452.808	450.437	chewRate_Tchewing total_chews_du avg_chewRate_Tmeal total_chews VAR_chewRate_perSeq
Bite	0.292	0.221	−39.669	266.946	−562.884	483.546	total_bite
Swallow	0.266	0.212	−0.698	210.725	−413.72	412.323	SD_ISF var_ISF total_swallow avg_swlRate_Tmeal
Sensor	0.342	0.42	−8.627	252.591	−503.706	486.452	avg_pwr_dB avg_Waveform_Length avg_pwr

All values are in g.

Note: SD, standard deviation; LOA, limit of agreement; features, features selected from forward selection.

**Table 2:** Model coefficients for mass intake models.

	Features	Coefficient	SE	t value	P value	Adj R2	Root MSE
Full	total_bite	10.951	1.022	10.731	<0.0001	0.924	211.6
	avg_pause_du	23.186	4.535	5.118	<0.0001		
	VAR_chews_du_perSeq	8.011	2.466	3.251	0.0020		
	VAR_chews_perSeq	−2.744	1.268	−2.166	0.0376		
	avg_Total_Entropy	−0.694	0.757	−0.921	0.3749		
Chew	chewRate_Tchewing	617.990	63.245	9.782	<0.0001	0.922	214.2
	total_chews_du	2.338	0.326	7.175	<0.0001		
	avg_chewRate_Tmeal	−621.501	123.337	−5.046	<0.0001		
	total_chews	−1.081	0.200	−5.421	<0.0001		
	VAR_chewRate_perSeq	−1181.820	605.355	−1.954	0.0588		
Bite	total_bite	15.056	0.520	28.971	<0.0001	0.885	259.1
Swallow	SD_ISF	5859.918	752.220	7.796	<0.0001	0.932	199.2
	var_ISF	−11453.700	1864.199	−6.151	<0.0001		
	total_swallow	2.805	0.559	5.031	<0.0001		
	avg_swlRate_Tmeal	−1581.440	681.174	−2.325	0.0285		
Sensor	avg_Waveform_Length	19.765	5.939	3.344	0.sss0020	0.898	244.4
	avg_pwr	1916.531	693.078	2.871	0.0154		
	avg_pwr_dB	−943.763	1141.817	−0.864	0.3954		

Note: SE, standard error; Adj R2, adjusted R square; Root MSE, square root of mean square error.

### Models performance for estimating energy intake

**Figure 2:**
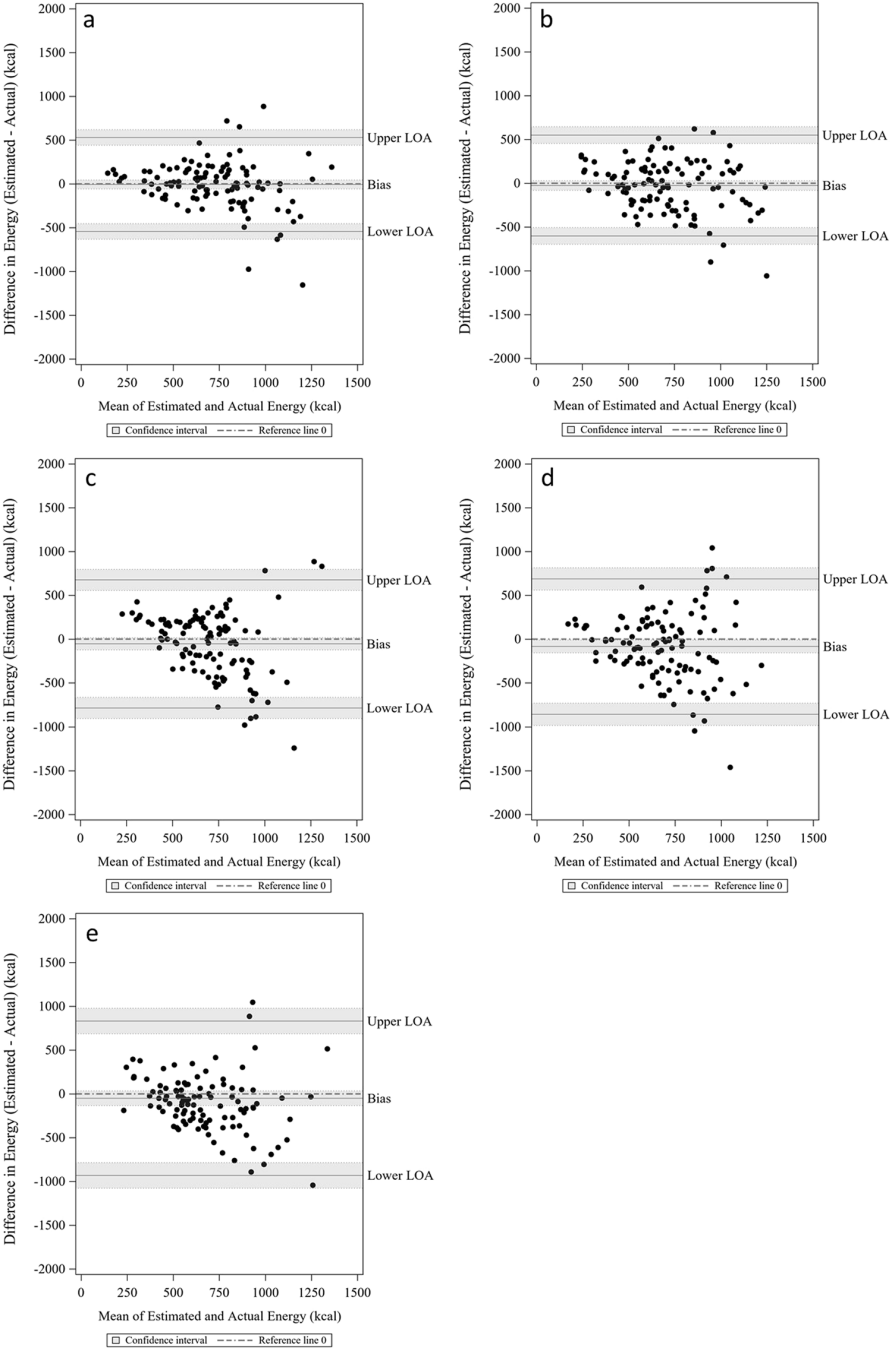
Bland-Altman plot of estimated and actual energy intake based on models. (**a**) full model; (**b**) chew model; (**c**) bite model; (**d**) swallow model; (**e**) sensor model.

Table 3 shows the mean and standard deviation of absolute percentage errors and biases for the full, chew, bite, swallow, and sensor models estimating the energy intake of a meal. Again, the full model had lowest mean absolute percentage error (mean = 0.301 kcal, SD = 0.338 kcal) and smallest bias (Mean = −6.095 kcal, SD = 273.75 kcal). The Bland-Altman plots also showed the narrowest limit of agreement (lower = −542.6 kcal, upper = 530.5 kcal), and the bias for full model was not significant since the confident interval covered 0 (Fig. 2a). Six independent features were selected to minimize mean absolute percentage error: Standard deviation of chewing duration per chewing sequence, Standard deviation of instantaneous swallow frequency, Standard deviation of number of chews in a chewing sequence, total pause duration, variance of amplitudes of sensor signals across all chewing bouts, and average pause duration per meal. The standard deviation of chewing duration had the most statistically significant coefficient (*t* value = 6.07, p < 0.0001) with about 198.7 kcal of energy intake increase for one unite increase in this feature (Table 4).

**Table 3:** Mean absolute percentage errors and mean errors for energy intake models.

	Relative reporting error		Mean estimation error		Lower LOA	Higher LOA	Predictors
	Mean	SD	Mean	SD			
	0.301	0.338	−6.095	273.75	−542.644	530.455	SD_chews_du_perSeq SD_ISF SD_chews_perSeq total_pause_du avg_var_Amplitude avg_pause_du
Chew	0.413	0.533	−25.932	293.763	−601.707	549.844	SD_chews_du_perSeq VAR_chews_perSeq
Bite	0.48	0.587	−53.795	372.226	−783.358	675.768	total_bite
Swallow	0.471	0.456	−84.113	393.468	−855.31	687.084	SD_ISF
Sensor	0.51	0.825	−49.798	449.729	−931.266	831.671	avg_zero_crossings avg_Spectral_energy

Note: SD, standard deviation; LOA, limit of agreement; features, features selected from forward selection.

**Table 4:** Model coefficients for energy intake models.

	Features	Coefficient	SE	t value	P value	Adj R2	Root MSE
Full	SD_chews_du_perSeq	198.705	32.796	6.070	<0.0001	0.899	250.8
	SD_ISF	1254.155	249.931	5.022	<0.0001		
	SD_chews_perSeq	−83.250	24.772	−3.372	0.0021		
	avg_var_Amplitude	29587.910	10688.730	2.785	0.0079		
	total_pause_du	0.460	0.167	2.768	0.0082		
	avg_pause_du	−10.364	9.147	−1.138	0.2717		
Chew	SD_chews_du_perSeq	232.011	17.166	13.528	<0.0001	0.872	283.1
	VAR_chews_perSeq	−6.561	1.319	−4.986	<0.0001		
Bite	total_bite	14.691	0.727	20.226	<0.0001	0.790	362.1
Swallow	SD_ISF	3390.149	184.261	18.410	<0.0001	0.757	389.6
Sensor	avg_zero_crossings	13.773	0.940	14.670	<0.0001	0.794	359.0
	avg_Spectral_energy	105577.600	23307.070	4.494	<0.0001		

Note: SE, standard error; Adj R2, adjusted R square; Root MSE, square root of mean square error.

## Discussion

We employed forward feature selection using absolute percentage error as the criterion to screen for useful features during our multi-factorial modeling process. We found that for both mass and energy intake, models which combine video observation features and sensor features presented the lowest absolute percentage errors with a mean underestimation of 7.7 g and 6.1 kcals for mass and energy intake per meal, respectively. The root MSEs of these models suggested that, for individuals, meals could be predicted within ±212 g and ±251 kcals, approximately 58% and 69% of actual intake. Because our models are derived from group eating characteristics (vs individual characteristics), and because a wide variety of foods was included, our models are expected to be more generalizable than those from previous studies. Our work, though carried out under controlled laboratory conditions, holds potential for predicting mass intake and energy intake in free living situations.

In our full models, which contained information from both the video observation and the wearable sensor, we found that bites, chews, and within-meal pauses were important for estimation of both mass and energy intake. The total number of bites was selected in the mass model and this was expected as initiating a bite is the first physical step of food intake. In this work, the bite was defined as an event when food as actually placed in the mouth, which is an important differentiator from hand gesture/wrist motion used in some studies^19^. The hand-to-mouth gestures (e.g. a gesture to use a napkin) may be confused with bites, therefore use of actual bites is potentially more accurate.

Chewing-related features selected by the full model were variation of chewing duration and number of chews per chewing sequence. Chewing is a reliable indicator of solid food intake and because sucking requires a jaw motion similar to that of chewing, could also potentially be used for liquid intake events that use sucking as the way of consumption (e.g. consuming liquids through a straw)^39^. Chewing duration and chew counts are influenced by the amount of food in the mouth and texture (rheological properties) of the food consumed and therefore chewing data provides valuable information about the mass, and, potentially, energy density of the food being consumed.

Within meal average pause duration was also found to be significant for predicting mass intake per meal. The pause duration is an indication of the eating rate and the environment/conditions in which food is being consumed (i.e. group setting or eating alone) and both have been found to have a relationship with the amount consumed. The study of^40^ investigated the effect of within meal pauses among 16 subjects and found that the subjects consumed more when there were within meal pauses compared to no pauses. Our results support the hypothesis that duration of within meal pauses has predictive power for estimation of mass and energy intake.

For chew only models, chewing rate, chew counts and chewing duration related features were selected by the forward feature selection procedure (Tables 1 and 3). The chewing rate varies between individuals and food properties such as composition, structure, volume, size and shape. Typically, hard foods (e.g. carrot, peanuts) would require more chews with higher chewing force compared with soft foods (e.g. cake). Chewing rate also contributes to eating rate and therefore is significantly correlated with the total amount of food consumed. A recent study^41^ showed that a faster rate of eating increased the mass and energy intake within a single meal. Similarly total chew counts per meal has been shown to have a relationship with total mass consumed in a single meal experiments^42^. We note that the method has proportional errors shown in Bland-Altman plots especially in the mass intake models using chew and sensor features. It may relate to the way that how foods were eaten. For example, people may eat larger piece of food with fewer chews than expected, which may cause underestimation of mass intake for those models based on chews and chewing-related sensor features. However, this is only a hypothesis since eating activity is complex and varies among individuals. Further studies are needed to test the hypothesis and investigate how food characteristics affect chews, bites and swallows features in order to improve our current models.

The bite model selected the total number of bites as the best predictor of mass intake. In the case of the swallow only model, total number of swallows, swallow rate, and instantaneous swallow frequency related features were selected. Since swallowing is necessary to consume food, we expected swallowing to be one of the most significant predictors of total intake. The frequency of swallowing significantly increases during food intake compared with spontaneous swallowing, and therefore, is a reliable indicator of ingestion.

Mass intake and energy intake models were predicted by comparable features in our chew, bite and swallow models. In energy intake full models, more swallow related features were selected and the bite features were not selected, whereas other selected features were similar. One potential reason could be related to the food items present in the meals comprising of both solid foods and caloric beverages. Swallows play an important role in all food intake related events irrespective of their liquid, semi-solid, or solid state whereas bites are not necessary for liquid ingestion. There is a positive relationship between number of swallows and the energy content of the ingested food. This relationship is poorly understood and requires further investigation.

Compared with our previously reported individual Counts of Chews and Swallows (CCS) models derived from the same dataset but using individual prediction methods, our current group models (participant independent) achieved similar accuracy (mean absolute percentage error 30.1% for current full model and 30.42% for CCS model by Fontana *et al*.^21^). Compared with the individual CCS model by Fontana *et al*., our energy intake mode has slightly wider limits of agreement (Lower LOA = −542.644; Upper LOA = 530.455; SD = 273.75 kcal) (Fig. 2). The models presented in this paper were built on all participants rather than calibrated to each individual participant which increases the generalizability and account for inter-person variability in eating habits.

One of our motivations is to use wearable sensors that carry lesser burden compared to other methods, including multimedia (image) diaries. Significant information may be obtained from sensors for features that describe eating behavior (such as eating rate) that are not available from images^24,25,43^. There are several other studies which have proposed use of sensor data for estimation of mass and energy intake. For example, the combination of audio and motion sensors with video annotation and ground truth food type achieved a mean absolute percentage error of 35.4% for solid food and 47.2% for liquid food^20^. Another relevant work used acoustic signals for bite weight estimation for only 3 food items where the prediction error varied from 19.4% to 31%^18^. However, in^18^, both the number of food items and the number of participants (8 participants, single visit) were small.

### Strengths and Limitations

The innovation of this study was exploration of features describing bites, chews and swallows during the meal in the context of their predictive ability to estimate mass and energy intake. Such analysis is potentially useful in considerations of sensor choices. In the present study, we tested the models on a wide range of foods with a variety of physical characteristics while in other studies the food variety is limited^12,18^. Another difference between this work and our previous work^34^ is that mass and energy intake estimation is on the meal level compared to the food item level. A meal usually consists of multiple foods and people often mix them during eating^44^. Although meal level estimation is more difficult than item level estimation because of the greater variability of the food present (in mass and energy content), our models results are comparable to previously reported results^21^. We further presented energy intake estimation models directly trained on the features extracted from the video and sensor signals without the need of first estimating mass. This approach is different from our previous study where mass and energy density of food (known food type) were used to compute energy intake^21^. The average percentage error in estimation of energy intake is close to the error for mass intake (30.1% compared to 25.2%). The estimates of ingested mass are potentially useful in combination with computer vision methods, which most frequently attempt to estimate volume (portion size) from a single pose image, which is an ill-posed problem unless special actions are taken by the user, such as placing a fiducial marker into the view of the camera. The wearable sensor may provide independent estimates of food portion size or refine image-based estimates. Similarly, the sensor-based estimates of energy intake may be used as points of comparison in field experiments where the ground truth for ingested energy is not available.

However, because our study was laboratory-based, the results cannot be directly extended to community-dwelling conditions. Therefore, future experiments will involve the testing of the models on data collected during multi-meal experiments collected in free living situations. Although video observation and annotation as used in this manuscript is highly accurate in counting chews, bites and swallows, we do not suggest it as a method to measure energy intake in practical situations, rather our approach is to use wearable sensors to detect and characterize bites, chews and swallows, potentially in combination with an egocentric wearable camera that could capture the foods being eaten. Also, the nature of the sensor (piezoelectric strain sensor) we used for monitoring chewing presented a limitation. The sensor was required to be attached to the skin via medical adhesive, which limited the long term use of the sensor. Since the sensor used can detect only chewing, sensor information was not available for liquids consumed by themselves (i.e., swallowing only events that do not require chewing). However, as the sensor technology develops, we are improving the sensors. For example, since the study was conducted, we have reported on user-friendly sensors not needing an adhesive attachment that can provide the same chewing information as the piezoelectric strain sensor used in this study^45^.

### Future Directions

Future studies will involve the evaluation of the proposed models in multi-day and multi-meal free-living experiments, where models will be trained to estimate mass and energy intake over several meal types (including breakfast, lunch, dinner and snacks within and among participants). This approach will ensure the practical usability of the developed models for application to real life eating events.

Methods need to be developed to determine food type and improve estimation accuracy. The accuracy of food type recognition can be substantially improved by employing imaging techniques such as wearable cameras. Potential directions include combining emerging computer vision methods to provide recognition of food type and energy density with sensor methods that provide independent estimates of mass and energy intake. The food intake recognition from analysis of the sensor signals may also be used to trigger a wearable camera during food consumption and thus provide a completely passive method for energy intake measurement. Our future work will add image recognition and sensor-based models.

## Supplementary information

Supplemental Figures and Tables

## Data Availability

The datasets generated during and/or analyzed during the current study are available from the corresponding author on reasonable request.
