# Supplementary material for: Statistical models for meal-level estimation of mass and energy intake using features derived from video observation and a chewing sensor
Source: Sci Rep. 2019 Jan 10;9:45. doi: 10.1038/s41598-018-37161-x (PMC6328599; doi:10.1038/s41598-018-37161-x)
Supplement: Supplementary file 1 — Supplemental Figures and Tables [file 41598_2018_37161_MOESM1_ESM.pdf]

Supplemental information

Statistical models for meal-level estimation of mass and energy intake using features derived from video observation and a chewing sensor

Xin Yang[1], Abul Doulah[2], Muhammad Farooq[2], Jason Parton[1], Megan A. McCrory[3], Janine A Higgins[4], Edward Sazonov[2]*

1. Institute of Business Analytics, University of Alabama, Tuscaloosa, AL, United States
2. Department of Electrical and Computer Engineering, University of Alabama, Tuscaloosa, AL, United States
3. Department of Health Sciences, Boston University, Boston, MA, United States
4. Department of Pediatrics, University of Colorado, Anschutz Medical Campus, Denver, CO, United States

*Corresponding: Edward Sazonov, Department of Electrical and Computer Engineering, University of Alabama, Tuscaloosa, AL, United States

Figure S1. Examples of foods provided during experiments.

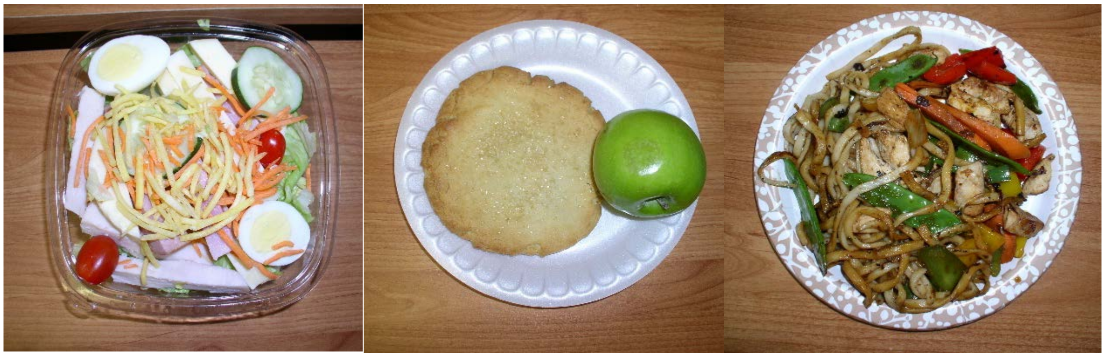

Figure S2. Microstructure of typical eating events.

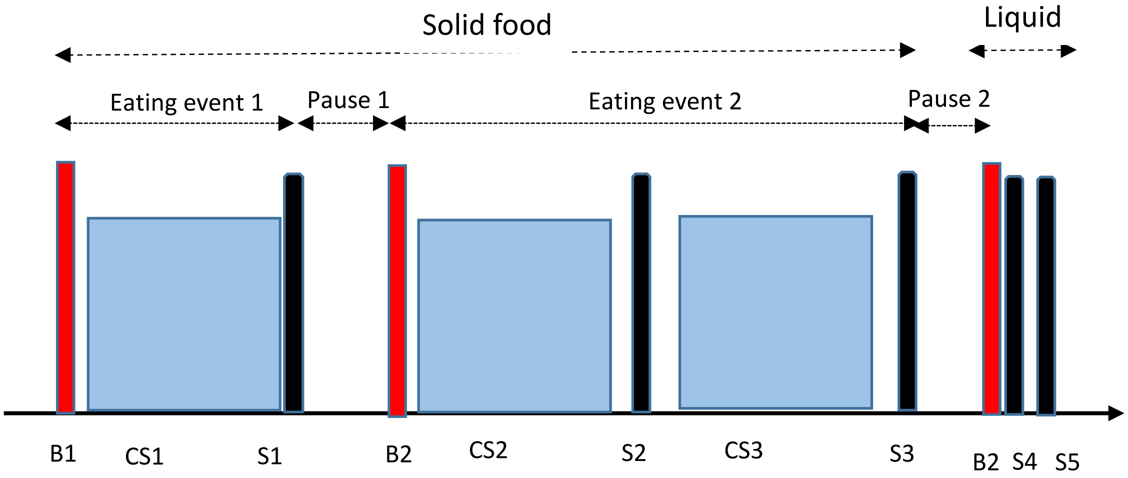

Note: B, bite; CS, chewing sequence; S, swallow.

Table S1. Variables obtained from video observations and the chewing sensor.

	Variable names	Description
		**Dependent variables**
	total_mass	Total mass per meal
	actual_energy_intake	Total energy intake per meal
		**Independent variables**
Chew	*chews_seq*	Number of chewing sequence per meal
	total_chews	Total chews number per meal
	avg_chews_perSeq	average chews number on chewing sequence per meal
	SD_chews_perSeq	Standard deviation of chews number on chewing sequence per meal
	VAR_chews_perSeq	Variance of chews number on chewing sequence per meal
	total_chews_du	total chew duration per meal
	avg_chews_du_perSeq	average chew duration on chew sequence per meal
	SD_chews_du_perSeq	Standard deviation of chew duration on chew sequence per meal
	VAR_chews_du_perSeq	variance of chew duration on chew sequence per meal
	avg_chewRate_perSeq	average chew rate on chew sequence per meal
	SD_chewRate_perSeq	Standard deviation chew rate on chew sequence per meal
	VAR_chewRate_perSeq	Variance chew rate on chew sequence per meal
	chewRate_Tchewing	Chew rate : total chew number per meal / total chew duration per meal
	avg_chewRate_Teating	Chew rate: total chew number/(bite duration + chew duration + swallow duration)
	avg_chewRate_Tmeal	Chew rate: total chew number/(bite duration + chew duration + swallow duration + pause duration)
Bite	*total_bite*	total bite number per meal
	avg_biteRate_Teating	Bite rate: total bite number/(bite duration + chew duration + swallow duration)

	avg_biteRate_Tmeal	Bite rate: total bite number/(bite duration + chew duration + swallow duration + pause duration)
	avg_IBF	Average instantaneous bite frequency
	SD_IBF	Standard deviation of instantaneous bite frequency
	var_IBF	Variance of instantaneous bite frequency
Swallow	*total_swallow*	Total swallow number per meal
	avg_swlRate_Teating	total swallow number/(bite duration + chew duration + swallow duration)
	avg_swlRate_Tmeal	total swallow number/(bite duration + chew duration + swallow duration + pause duration)
	avg_swl_bite	Average swallow number between bites
	SD_swl_bite	Standard deviation of swallow number between bites
	var_swl_bite	Variance of swallow number between bites
	avg_ISF	Average instantaneous swallow frequency
	SD_ISF	Standard deviation of instantaneous swallow frequency
	var_ISF	Variance of instantaneous swallow frequency
Pause	*total_pause_du*	Total pause duration per meal
	avg_pause_du	Average pause duration per meal
	SD_pause_du	Standard deviation of pause duration per meal
	var_pause_du	Variance of pause duration per meal
Sensor	*avg_Total_mean_Amplitude*	Sum of the average amplitude of chewing bouts/sequences
	avg_Total_pwr	sum of the signal power of all chewing bouts
	avg_Total_pwr_dB	sum of the signal power of all chewing bouts represented in dB
	avg_Total_short_period	summation of the chewing period of all the chewing bouts. Periods were computed based on the peaks of autocorrelation function.
	avg_Total_std_Amplitude	
	avg_Total_var_Amplitude	
	avg_mean_Amplitude	mean signal amplitude across all the chewing bouts in a meals
	avg_pwr	average of the power across all the chewing bouts in a meal.

avg_pwr_dB	average of the power across all the chewing bouts in a meal, in dB
avg_short_period	average of the chewing period of all the chewing bouts. Periods were computed based on the peaks of autocorrelation function.
avg_std_Amplitude	standard deviation of amplitudes across all chewing bouts
avg_var_Amplitude	variance of amplitudes of sensor signals across all chewing bouts
avg_Entropy	average entropy of the chewing bouts
avg_zero_crossings	average number of zero crossings in all the chewing bouts
avg_mean_time_ZeroCrossing	average time between the zero crossing in across all chewing bouts in a meal
avg_Waveform_Length	average waveform length of chewing bouts
avg_Spectral_energy	average spectral energy of the chewing bouts.
avg_Total_Entropy	sum of the entropies of the chewing bouts in a meal
avg_Total_zero_crossings	sum of all the number of zero crossings in all the chewing bouts
avg_Total_mean_time_ZeroCrossing	sum of the time between the zero crossing in across all chewing bouts in a meal
avg_Total_Waveform_Length	sum of the waveform lengths of all chewing bouts
avg_Total_Spectral_energy	sum of the spectral energies of the chewing bouts.
avg_Max_frequency	average frequecy of the frequencies with the highest contribution to the frequency spectrum
